# Regenerative medicine approaches for the management of respiratory tract fistulas

**DOI:** 10.1186/s13287-020-01968-1

**Published:** 2020-10-23

**Authors:** Angelo Trivisonno, Dania Nachira, Ivo Boškoski, Venanzio Porziella, Giuliana Di Rocco, Silvia Baldari, Gabriele Toietta

**Affiliations:** 1 Department of Surgical Science, University of Rome “La Sapienza”, Viale Regina Elena 324, 00161 Rome, Italy; 2 Department of General Thoracic Surgery, Fondazione Policlinico Universitario Agostino Gemelli IRCCS, Largo A. Gemelli 8, 00168 Rome, Italy; 3 Digestive Endoscopy Unit, Fondazione Policlinico Universitario Agostino Gemelli IRCCS, 00168 Rome, Italy; 4 Department of Research, Advanced Diagnostic, and Technological Innovation, Translational Research Area, IRCCS Regina Elena National Cancer Institute, via E. Chianesi 53, 00144 Rome, Italy

**Keywords:** Adipose tissue, Fistula, Regenerative medicine, Respiratory tract, Lipotransfer, Mesenchymal stromal cells, Head and neck, Tracheoesophageal fistula, Minimally invasive treatments, Airway defects restoration

## Abstract

Respiratory tract fistulas (or fistulae) are abnormal communications between the respiratory system and the digestive tract or the adjacent organs. The origin can be congenital or, more frequently, iatrogenic and the clinical presentation is heterogeneous. Respiratory tract fistulas can lead to severely reduced health-related quality of life and short survival. Therapy mainly relies on endoscopic surgical interventions but patients often require prolonged hospitalization and may develop complications. Therefore, more conservative regenerative medicine approaches, mainly based on lipotransfer, have also been investigated. Adipose tissue can be delivered either as unprocessed tissue, or after enzymatic treatment to derive the cellular stromal vascular fraction. In the current narrative review, we provide an overview of the main tissue/cell-based clinical studies for the management of various types of respiratory tract fistulas or injuries. Clinical experience is limited, as most of the studies were performed on a small number of patients. Albeit a conclusive proof of efficacy cannot be drawn**,** the reviewed studies suggest that grafting of adipose tissue-derived material may represent a minimally invasive and conservative treatment option, alternative to more aggressive surgical procedures. Knowledge on safety and tolerability acquired in prior studies can lead to the design of future, larger trials that may exploit innovative procedures for tissue processing to further improve the clinical outcome.

## Introduction

Fat grafting, referred also as lipotransfer, involves harvesting of adipose tissue, processing of the collected fat to eliminate oil, liposuction fluids, and blood components, and then re-injection of the manipulated tissue into the area that needs treatment [1]. The first documented surgical fat grafting procedure dates back to 1893 when Gustav Neuber described the transfer of adipose tissue harvested from the forearm into the periorbital region to correct a depressed scar [2]. In 1987, Sydney R. Coleman developed an innovative technique of liposuction allowing for adipose tissue harvest under local anesthesia with less extensive damage [3]. The procedure of fat grafting has been broadly explored to repair soft tissue volume loss (reconstructive surgery) and to enhance cosmetic appearance (cosmetic surgery) [3, 4]. More recently, fat grafting has also been used to promote tissue or organ healing (regenerative medicine) [1, 5–7]. Several parameters such as fat preparation, implantation techniques, and recipient site may affect graft retention [8]. As a result, in the absence of a general consensus on a standardized procedure, the clinical outcome of lipotransfer is not always predictable [9, 10].

In 2001, Zuk et al. demonstrated the presence within the adipose tissue of multipotent cells able to differentiate in vitro into adipogenic, chondrogenic, myogenic, and osteogenic cells [11]. This discovery provided further support for the perspective use of adipose tissue-derived material for regenerative purposes [12, 13]. The isolation of multipotent cells from the adipose tissue complex involves several steps: (1) fat digestion by a solution containing collagenase, (2) elimination of tissue debris by filtration, (3) centrifugation to collect the cellular component of the so-called stromal vascular fraction (SVF), (4) expansion of the isolated cells in culture to obtain adipose tissue-derived mesenchymal cells, and (5) flow cytometry analysis for phenotypic characterization of the isolated cells [14]. According to the definition released by the International Federation for Adipose Therapeutics and Science (IFATS) and the International Society for Cellular Therapy (ISCT), uncultured SVF cells are a heterogeneous population that includes stromal cells, endothelial cells, erythrocytes, fibroblasts, lymphocytes, monocyte/macrophages, and pericytes [15, 16]. Mesenchymal stromal/stem cells (MSC), referred also as adipose tissue-derived stromal cells (ASC), are characterized by rapid plastic adherence in culture; moreover, they express the phenotypic markers CD90, CD73, CD105, and CD44, while they are negative for CD45 and CD31 expression; in addition, MSC can differentiate into osteocytes, adipocytes, and chondrocytes in vitro in the presence of appropriate inductive media [15]. During the course of the years, adipose tissue-derived multipotent cells [11] were named also as stem cells [17], as mesenchymal stromal cells [15] and, more recently, as medicinal signaling cells [18], maintaining the MSC acronym [16]. The evolution of the nomenclature reflects a paradigm shift on how MSC are believed to exert their therapeutic effect in regenerative medicine procedures. In facts, the term “multipotent stem cells” was originally coined to imply that MSC might differentiate into cells which directly participate into tissue healing (building block activity). Several experimental and clinical evidences subsequently indicated that, despite the development of different strategies aiming at improving cell engraftment [19], the number of cells which are actually able to survive and persist upon transplant, to differentiate in vivo and to take part in tissue regeneration are far too low to justify the clinical benefit observed in cell therapy procedures [20]. Therefore, the attention was pointed to the ability of MSC, as “medicinally signaling cells”, to produce trophic, immunomodulatory factors, either directly or via extracellular vesicles, which might promote tissue regeneration and/or tissue stem cells homing (paracrine activity) [21]. The exact molecular mechanism(s) underlying the regenerative potential associated with adipose tissue- and cell-based therapies still require complete elucidation [22]. MSC are believed to exert their pro-healing function mainly through the release of paracrine factors and extracellular vesicles that may stimulate the migration and activation of local tissue-specific stem cells that contribute to tissue regeneration, promotion of neo-angiogenesis, modulation of inflammatory and immunomodulatory responses, and increase of anti-oxidative and anti-apoptotic effects [23, 24]. Several regenerative medicine clinical trials using MSC cell transplant procedures have been performed [25]; in particular, treatment of perianal fistulising Crohn’s disease, a chronic inflammatory disorder of the gastrointestinal tract, using cell therapy has been extensively investigated in virtue of the immunomodulatory properties of MSC [26–28]. A phase III study verified the safety and the efficacy in long-term closure of perianal fistulas by local injection of adipose tissue-derived MSC [29].

Placement of esophageal stent [30] or bioprosthetic materials [31] are currently used for the management of different esophago-respiratory fistulas. Unfortunately, this kind of surgical intervention often requires long hospitalization and may be associated with a considerable risk of adverse events. Recently, the therapeutic efficacy of the delivery of cell and tissue-based products for the treatment of fistulas of different etiology has been studied. We performed a narrative literature review on the management of different kinds of fistulas and esophageal and airway defects through the administration of cellular and tissue-based products, as a conservative alternative procedure to more aggressive surgery. We then focus on the possible future directions, including the potential use of different methods of adipose tissue manipulation, which may provide an opportunity to improve the clinical outcome of the procedure. In order to identify the studies evaluating the effects of autologous fat grafting and/or mesenchymal stromal cell therapy on airway tissue defects, we interrogated PubMed, Web of Science, Scopus, and Google Scholar electronic databases. Moreover, we consulted the ClinicalTrials.gov trial registry. We conducted literature search by combining Medical Subject Headings terms such as “respiratory tract fistula", “mesenchymal stromal cell”, “adipose tissue-derived stromal cells”, “stromal vascular fraction”, “lipoaspirate”, and “adipose tissue”. Studies were not constrained by publication date or publication status. Only clinical studies written in English were examined. Identified articles were mainly case reports since no clinical study with a large sample size has been evaluated so far (Table 1). Moreover, the nature of the disease, as well as the method of processing and local administration of the material, predominantly derived from autologous adipose tissue, varied among the studies.

**Table 1:** Reports of therapeutic procedures involving fat or mesenchymal stromal cells for the management of respiratory tract fistulas

Condition	Intervention	Patients enrolled	Reference
Oroantral fistula	Autologous buccal fat pad	1+ 25	[32–34]
Pharyngocutaneous fistula	Autologous fat	1 + 1	[35, 36]
Tracheoesophageal fistula	Autologous fat	1	[37]
Tracheomediastinal fistula	Autologous adipose tissue SVF in fibrin glue	1	[38]
Bronchopleural fistula	Autologous adipose tissue-derived MSC-seeded matrix graft	1	[39]
	Autologous bone marrow-derived MSC	1 + 2	[40, 41]
	Umbilical cord MSC	1	[42]
	Autologous fat	8	[43]

*SVF* stromal vascular cells (uncultured), *MSC* mesenchymal stromal cells

## Clinical application of adipose tissue-derived material for the treatment of respiratory tract fistulas

According to the Medical Subject Headings (MeSH) definition, a respiratory tract fistula is “an abnormal passage communicating between any component of the respiratory tract or between any part of the respiratory system and surrounding organs”. If left untreated, respiratory tract fistulas are associated with high mortality rates [44–46]. The most common interventional therapy relies on stent placement. In addition, more conservative strategies based on regenerative medicine approaches have also been considered. In the following sections, we briefly review some of the tissue/cell-based clinical studies described for the management of various types of respiratory tract fistulas (Table 1).

### Oroantral fistula

An oroantral fistula (OAF) is a pathologic communication between the oral and the antral cavities. The removal of the maxillary posterior teeth is considered the major cause of OAF development. Small-size OAF tend to heal spontaneously, while surgical intervention is recommended for fistulas larger than 3 mm. Taking in consideration of the size of the OAF and the condition of the surrounding tissues, different therapeutic approaches have been evaluated [47]. Larger defects, such as the ones subsequent to tumor resection, may require the use of autogenous bone and soft tissue grafts, the placement of allogenous materials or xenografts. Flaps utilizing local tissue, such as buccal and palatal flaps, can be used to close moderate-sized defects. In particular, application of the buccal fat, a lobulated form of adipose tissue, has been quite extensively utilized since its description in 1977 [32–34]. The adipose tissue used to repair OAF is generally coated by the surrounding mucosa in 4 to 6 weeks, thus promoting complete epithelialization of the treated area [48].

### Pharyngocutaneous fistula

Pharyngocutaneous fistula (PCF) is a pathological communication involving the digestive tract and the skin of the neck. PCF is a quite common complication after head and neck surgery [49]. Presence of PCF may prolong recovery and delay adjuvant oncologic treatments. The majority of cases are treated with conservative management in order to promote spontaneous healing, but approximately 30% of patients require a more aggressive surgical intervention. Two different case reports have described successful PCF healing by fat grafting in patients undergone to partial pharyngectomy [35, 36]. In particular, in the case report described by Hespe et al., two rounds of autologous fat grafting delivered into the area immediately surrounding the PCF using both blunt cannulas and 18 gauge needles were performed to achieve complete fistula healing [36]. Conversely, Sapundzhiev et al. reported a case report of a patient administered with autologous fat around the internal opening of the PCF with a Peretti angular injection cannula using an endoscopic access to the neopharynx [35].

### Tracheoesophageal fistula

**Fig. 1:**
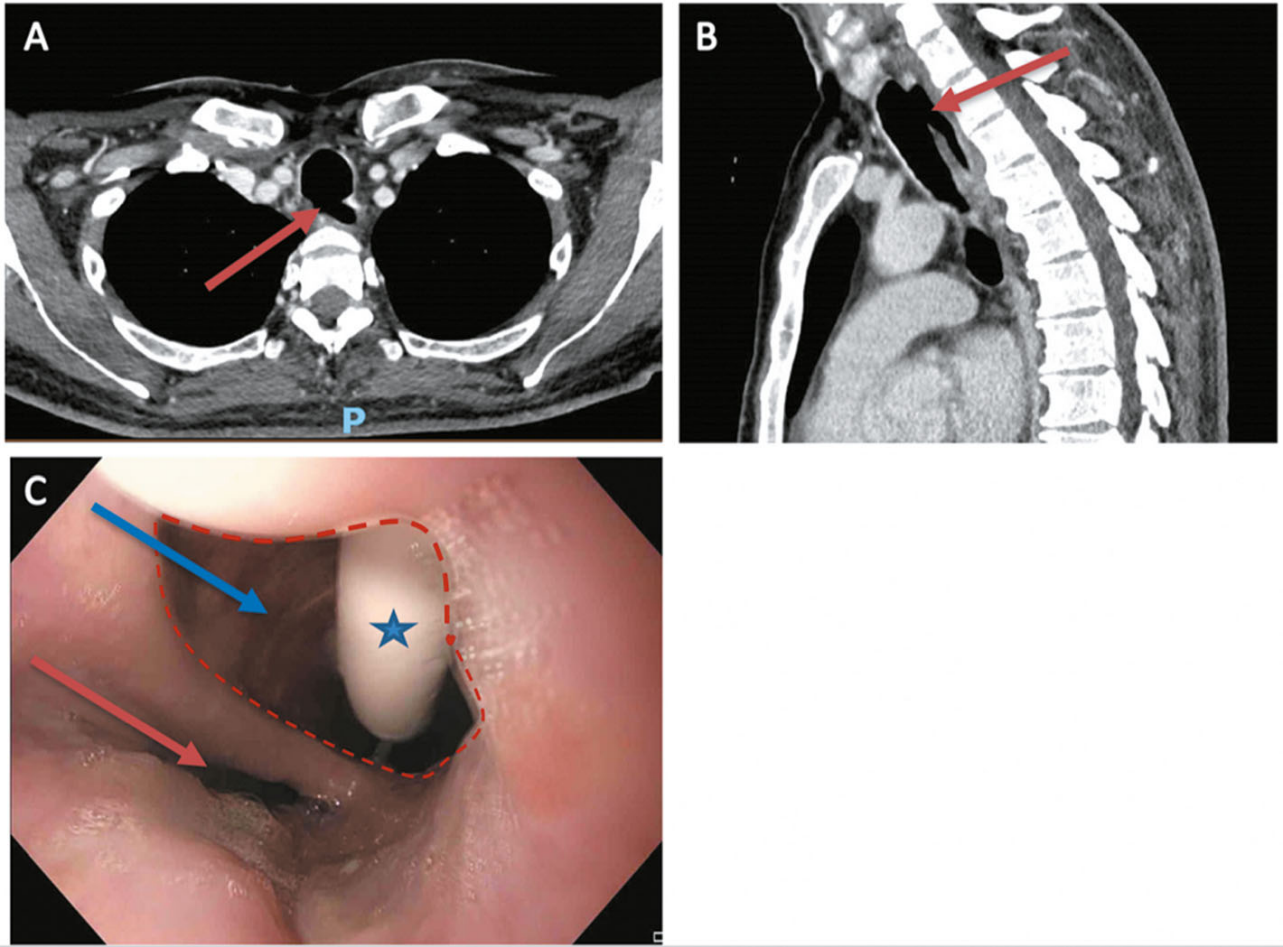
Iatrogenic tracheoesophageal fistula after emergency orotracheal intubation. Computed tomography (CT) images showing a large tracheoesophageal fistula (red arrow) in axial (**a**) and sagittal plane (**b**). Endoscopic image (**c**) of the fistula (red dashed line) between esophageal lumen (blue arrow), with a nasogastric tube inside (blue star), and tracheal lumen (red arrow)

Tracheoesophageal fistulas (TEF) are connections between the airway and upper gastrointestinal tract; they need prompt identification and treatment to prevent recurrent and intractable infections due to tracheobronchial contamination. TEF are broadly categorized into congenital and acquired fistulas, the latter group being further divided into nonmalignant and malignant. Congenital TEF occur in 1 in 3000–5000 live births [50], and they are usually diagnosed within the first year of life, while presentation in adults is rare [51]. Acquired nonmalignant TEF are mainly associated with traumatic injury, foreign body or caustic ingestion [52]. The majority of acquired nonmalignant TEF are mostly due to compression from an inflated endotracheal or tracheostomy tube cuff which may occur in approximately 0.5% of patients undergoing tracheostomy or intermittent positive pressure ventilation [53] (Fig. 1). Occasionally, nonmalignant acquired TEF may arise from local inflammation and infection, such as tuberculosis and granulomatous infection [54].

**Fig. 2:**
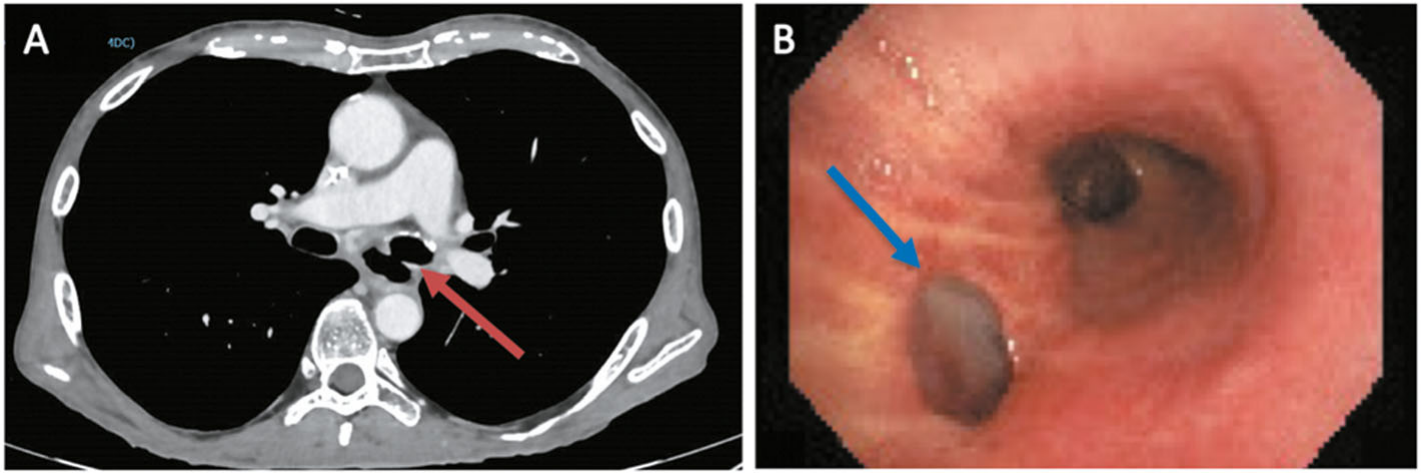
Neoplastic bronchoesophageal fistula after inductive radiotherapy. **a** CT image of the fistula between esophageal lumen and left main bronchus (red arrow). **b** Bronchoscopic view of the same fistula (blue arrow) on the membranous side of left main bronchus

Malignant acquired TEF have been associated with several types of cancers. In particular, TEF incidence has been reported as 4.5% following primary malignant esophageal tumors and 0.3% in primary malignant lung tumors [55]. Tumor invasion and cancer-related tissue necrosis may contribute to the pathogenesis of malignant TEF. In addition, also chemoradiotherapy and anti-angiogenic therapy, affecting local architectural and vascular tissue changes, can increase the risk of TEF formation [46] (Fig. 2). In this regard, cell-based therapies may attenuate chemotherapy-induced tissue injuries [56].

Small size TEF may close spontaneously, while fistulas over 20 mm in size are associated with poor survival [57]. Therefore, prompt therapeutic intervention is needed in order to arrest the contamination of the airway and enabling normal oral alimentation. Different approaches have been developed for the management of both acquired non-malignant [57] and malignant TEF [44, 46, 55]. Surgical interventions include esophageal stent placement, bypass, resection, and surgical repair. Conservative treatments, alternative to surgical procedures, mainly consist of supportive care to prevent contamination of the respiratory tract. Moreover, use of autologous tissue-assisted regenerative procedure may represent a valuable therapeutic option. In this regard, it has been described a case report of a 55-year old man affected by congenital TEF successfully treated with local injection of autologous fat using a pressurized injection device [37]. Long-term complete healing was observed after two sessions of administration of autologous fat and the patient remained asymptomatic more than 10 years.

### Tracheomediastinal fistula

**Fig. 3:**
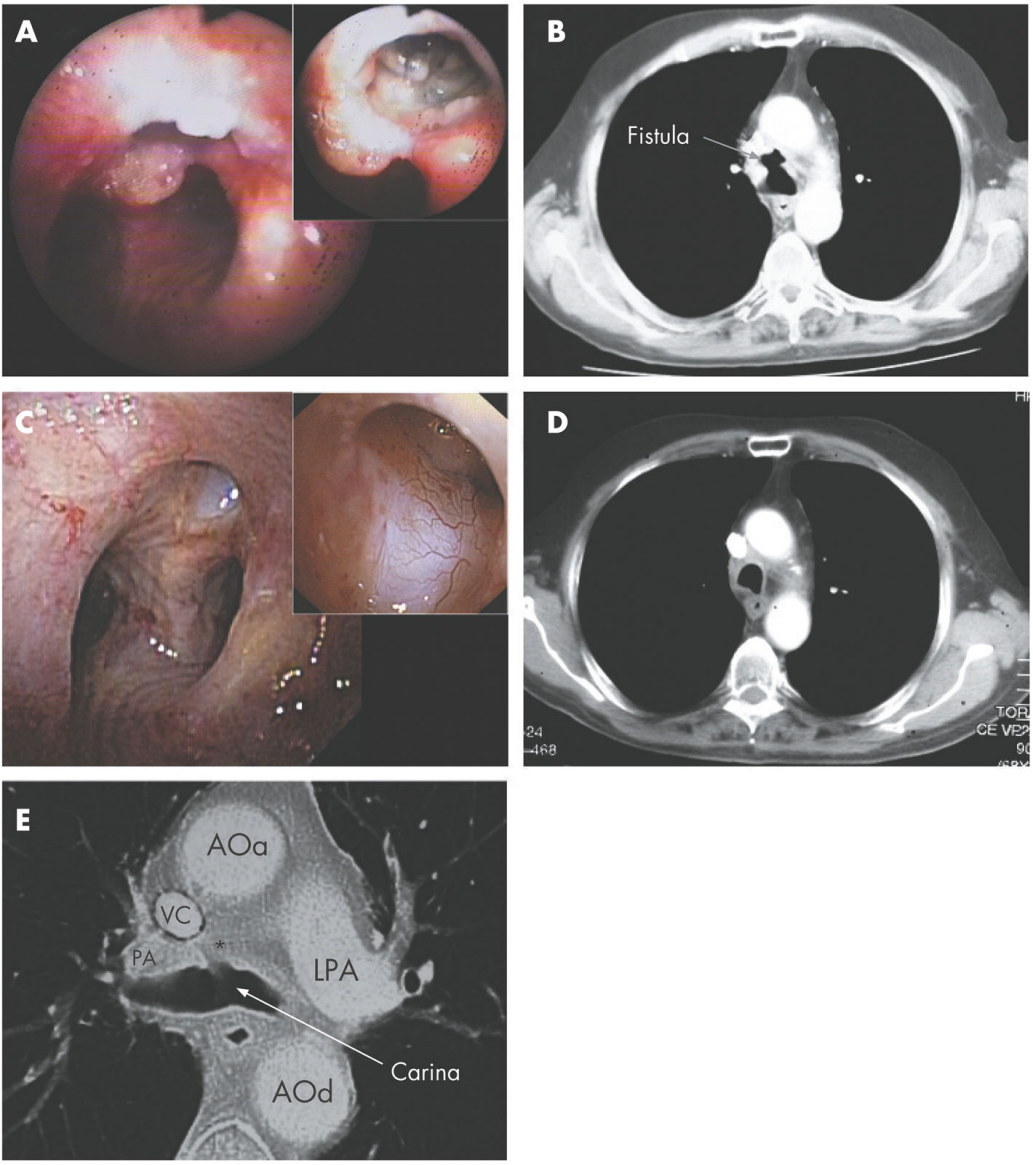
Bronchoscopic and CT images from the region of the fistula. **a** Bronchoscopic image recorded before cell therapy. The fistula can be seen on the anterior tracheal wall which had been totally destroyed after the laser treatment of the tumor. The entrance was about 10 mm in diameter and the bronchoscope could pass through it. Inset: Anthracotic mediastinal lymph nodes as seen through the wall of the fistula. **b** CT image recorded before cell therapy. The fistula was situated between the trachea and a pretracheal mediastinal cavity with an area of 2 cm^2^, next to the superior vena cava and pulmonary artery, near the ascending aorta. **c** Bronchoscopic image recorded 1 year after cell therapy. The entrance to the fistula was much smaller (diameter 3–4 mm). Inset: The walls of the fistula were covered with “new” epithelium and vessels as a result of neovascularisation and epithelialisation. **d** CT image from the same region of the fistula 1 year after cell therapy. One year after treatment the cavity had disappeared. **e** CT image from the region of the fistula recorded 1 year after cell therapy. This image is the only one to show remnants of the previous fistulous tract. It is clear that the fistula had closed. *, small depression; VC, superior vena cava; AOa, ascending aorta; AOd, descending aorta; PA, right pulmonary artery; LPA, left pulmonary artery. Reproduced with permission from Díaz-Agero Álvarez et al. [38]

A tracheomediastinal fistula (TMF) is a communication between the trachea and the mediastinum. TMF formation is rare and generally associated with airway tumors. Díaz-Agero Álvarez described a case report of TMF, subsequent to endoscopic laser therapy of tracheal cancer, treated with bronchoscopic administration of autologous ASC in fibrin glue suspension [38]. In particular, autologous ASC were isolated by collagenase digestion from 150 ml of lipoaspirate. Then, approximately 5.0 × 10^6^ cells were mixed in fibrin glue and injected through a bronchofibroscope into the cavity of a 2-cm^2^ TMF. One-year follow-up showed complete closure of the fistula with re-epithelialization and neovascularization of the area (Fig. 3) [38].

### Bronchopleural fistula

**Fig. 4:**
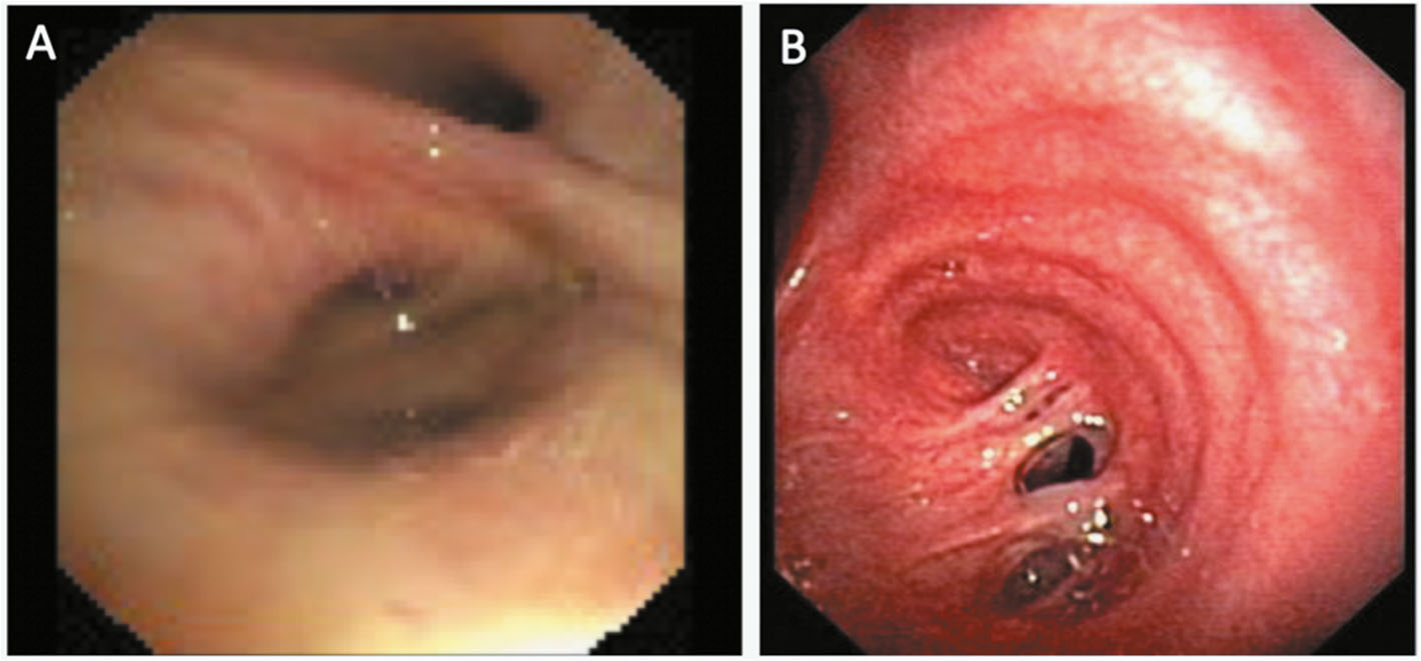
Bronchopleural fistulas after pulmonary lobectomy and pneumonectomy for lung cancer. **a** Bronchoscopic view of the fistula (and muco-purulent secretions) in the inferior right bronchial stump; **b** endoscopic view of the bronchial stump fistula after right pneumonectomy

A bronchopleural fistula (BPF) is defined as a pathological communication between the bronchial tree and the pleural space [58]. BPF is a severe postoperative complication of pneumonectomy or other pulmonary resection interventions with high rates of morbidity and mortality (Fig. 4). Therefore, surgical or bronchoscopic interventions are needed to promote BPF closure.

As conservative alternative to more traumatic surgical procedures, administration of mesenchymal cells has been performed in order to promote healing of the tissue surrounding the fistula [42, 59]. In particular, Petrella et al. described an approach of autologous bronchoscopic perilesional transplantation of ten million bone marrow-derived mesenchymal cells for the treatment of a small-caliber (3 mm) BPF developed in a 42-year-old man after right extra pleural pneumonectomy for malignant mesothelioma [40]. Aho et al. described a case report of a 66-year-old patient with a large (1.5 cm) BPF treated with a matrix graft seeded with autologous mesenchymal stem cells. Cells were obtained by collagenase digestion from autologous adipose tissue and underwent three passages of amplification in vitro, and finally, 2.5 × 10^7^ MSC were seeded on a matrix of synthetic bio-absorbable poly(glycolide:trimethylene carbonate) copolymer under Good Manufacturing Practices (GMP) procedures. Five days after cell seeding, the matrix graft was surgically placed over the BPF to promote healing [39]. The treated patient remained asymptomatic during the clinical follow-up of 1.5 years. Díaz-Agero Álvarez et al. described the treatment of two patients suffering from BPF by bronchoscopic administration of adipose tissue-derived stromal cells (ASC) isolated by collagenase digestion and not expanded in culture [41]. One patient, affected with a 6-mm diameter BPF, was administered with 4.0 × 10^6^ ASC leading to 80% closure of the fistula. Six months later, the procedure was repeated with the administration of additional 5.0 × 10^6^ ASC to achieve full healing. The second patient, who had a 3-mm diameter fistula, received 1.3 × 10^7^ ASC in a single procedure. Patients were observed for a 3-year follow-up, and no treatment-related adverse effects were reported [41]. Recently, Zeng et al. described a case report of successful closure of a BPF (5 × 2 mm) resulting from lobectomy, treated by administration through a flexible bronchoscope of 2.0 × 10^7^ umbilical cord MSC around the fistula [42]. A computed tomography scan performed 6 months after the treatment revealed fistula healing and the BPF did not relapsed during the 2-year follow-up. A different approach has been evaluated by Huramoto et al. in lung cancer patients undergoing lobectomy. The authors suggest that the use of isolated pericardial fat tissue to close the bronchial stump might prevent the occurrence of BPF [60]. Recently, endoscopic administration of autologous fat was performed for the treatment of BPF in 8 patients and resolution was observed in all cases [43].

## Clinical application of adipose tissue-derived material to promote tissue regeneration in the oropharyngeal tract

In the next sections, we present a brief overview of the studies of reconstructive/regenerative surgery assessing local administration of fat or adipose tissue-derived mesenchymal stromal cells to restore tissue loss or damage in the oropharynx (Table 2).

**Table 2:** Reports of therapeutic procedures involving fat or adipose tissue-derived cells to promote tissue regeneration in the oropharyngeal tract

Condition	Intervention	Patients enrolled	Reference
Tracheoesophageal puncture	Autologous fat	10	[61]
Hypertrophic tracheostomy scar	Autologous fat	10	[62]
Radiation-induced fibrosis and volume defects in head and neck oncology	Autologous fat	38 + 11 + 12	[63–65]
Velopharyngeal insufficiency	Autologous fat	11 + 251	[66, 67]
Vocal fold scars	Autologous SVF	8 + 1	[68, 69]
	Autologous fat	24	[70]
	Autologous nanofat and microfat	7	[71]
Unilateral laryngeal nerve paralysis	Autologous fat	> 90	[70, 72–75]

*SVF* adipose tissue-derived stromal vascular fraction cells (uncultured)

### Tracheoesophageal puncture

Tracheoesophageal puncture (TEP) with voice prosthesis placement is an extensively used technique to restore vocal function in patients undergoing total laryngectomy and pharyngolaryngectomy. One of the most frequent complications of this procedure, which usually requires to replace the voice prosthesis, is enlargements of the puncture, with leakage of saliva or food [76]. Administration of autologous fat around the puncture has been described as an effective and safe procedure, which allows the conservation of the voice prosthesis, by promoting the increase of the thickness of the tracheoesophageal wall [61]. In particular, 4 out of the 10 treated patients maintained long-term (up to 65 months) tracheoesophageal speech with no leakage.

### Hypertrophic tracheostomy scar

Hypertrophic scar formation at the site of tracheostomy is quite frequent. The scar tissue may attach to the trachea causing discomfort during the act of swallowing. Several surgical options have been described for hypertrophic scar ablation [77]. Fat, adipose tissue-derived mesenchymal stromal cells, and stromal cell-derived factors possess antifibrotic functions which exert a positive role in difficult scar treatment [78]*.* A minimally invasive procedure for the treatment of post-tracheostomy hypertrophic scar by means of intralesional administration of adipose tissue has been employed by Mazzola et al. resulting in valuable cosmetic results and in improved skin quality and texture [62]*.* All 10 treated patients enrolled in the study achieved satisfying esthetic and functional improvements.

### Head and neck reconstruction after radiotherapy

Reconstructive surgery may be required for functional and cosmetic soft tissue restoration in patients with head and neck cancers. Actually, treatment of head and neck cancers may require the surgical removal of a large amount of tissue surrounding the tumor; moreover, adjuvant therapeutic irradiation may result in extensive tissue damage and induction of radiation-induced skin fibrosis. Administration of autologous fat has been evaluated as a suitable method to achieve both esthetic and functional reconstruction in head and neck oncologic patients, partially restoring volume loss, reducing excessive scar formation and radiation-induced skin fibrosis in the treated areas [63–65, 79]. More than 60 patients have been treated using autologous fat administration in three different studies [63–65, 79]. Some concerns have been raised about the possibility of administered fat to promote residual tumor cell invasion and metastasis [80]. Preliminary data obtained using head and neck cancer cell lines both in vitro and in vivo suggest that the procedure may be safe, but further investigation performed on patient-derived tumor samples is needed [81].

### Velopharyngeal insufficiency

Velopharyngeal insufficiency (VPI) occurs when there is incomplete velopharyngeal closure. As reviewed by Nigh et al. administration of autologous fat has been collectively performed for the management of VPI in more than 250 patients [66]. Recently, the procedure was described for additional 11 adult patients [67]. Based on these preliminary studies, the procedure of injection pharyngoplasty with autologous fat could be considered as a safe and effective treatment option for mild VPI.

### Vocal fold scars

Vocal fold scars are scarring and fibrotic formations on the layer of the vocal cord. Preclinical studies support the rationale for using cell therapy for the treatment of vocal fold scarring which may occur as a result of surgical or iatrogenic injury [82]. A clinical team at the Hopitaux De Marseille, France, promoted a clinical trial entitled “Innovative treatment for scarred vocal cords by local injection of autologous stromal vascular fraction” (NCT02622464), described the first clinical case report [68], and have recently published the results on additional 8 patients [69]. The therapeutic intervention consisted in autologous adipose tissue harvest, enzymatic digestion, isolation of ASC under GMP conditions, and same-day local administration at the laryngeal level of 2.2–13.6 × 10^6^ viable uncultured cells. Follow-up analysis performed at 12 months indicated an improvement in the voice handicap index score without serious adverse events causally related to the treatment. In addition, Cantarella et al. described the results of the injection of approximately 0.2 to 3 ml of autologous fat in 24 patients with vocal fold scarring [70]. More recently, the same authors described the treatment of 7 patients with vocal fold scarring by nanofat and microfat grafting [71]. In particular, in this group of patients, microfat was administered deeply in the vocal fold and nanofat emulsion was injected in the most superficial layer of the vocal fold in the scarred tissue. Follow-up analysis performed at 3 months indicated improvements in the voice handicap index.

### Unilateral recurrent laryngeal nerve paralysis

Unilateral recurrent laryngeal nerve paralysis may occur secondary to injury of the recurrent laryngeal nerve due to cancers, trauma, and surgery. In 1991, Mikaelian et al. published a preliminary report describing a procedure of autologous fat injection into a paralyzed vocal cord in 3 patients affected by unilateral vocal cord paralysis [72]. Since then the procedure has been performed on several additional cases describing long-term (> 1 year) improvement of vocal parameters after a single fat injection [70, 73–75, 83, 84]. In particular, in a clinical trial (NCT02904824), a group of patients was treated by administration of adipose tissue and a second group with the same amount of adipose tissue in presence of a not-well quantified amount of ASC (cell-assisted lipotransfer) [74]. However, the study was inconclusive in determining any definite difference between the clinical outcomes of the two groups [74].

## Future directions

We have summarized in Tables 1 and 2 a number of published clinical studies on promotion of tissue healing in the respiratory tract applying a conservative, regenerative medicine-based approach. Most of the evidence-based data have been collected from single case reports or series of case studies. As a matter of facts, the design of larger, multisite clinical trials is hampered by the relatively small number of affected individuals that can be enrolled and by the lack of clinical institutions which have sufficient knowledge and resources for innovation implementation in this field***.*** Accordingly, the number of clinical trials which have been performed or are currently ongoing is still limited (Table 3). Bone marrow, adipose tissue, and umbilical cord blood are the most frequently utilized sources of MSC for clinical trials [23], including the ones described for fistula healing and tissue regeneration in the oropharynx (Table 3). Bone marrow and adipose tissue are replenishable sources of MSC suitable for autologous transplant [85]. Bone marrow collection is an invasive procedure, while subcutaneous adipose tissue can be easily harvested [86]. Adipose tissue contains up to 500 more MSC cells than an equivalent amount of bone marrow; moreover, adipose tissue-derived MSC can be easily expanded in vitro since they have higher proliferation rate compared to bone marrow-derived MSC [87]. In addition, MSC derived from adipose tissue promote stronger immunosuppressive effects than MSC isolated from other sources [88]. MSC in umbilical cord are rare but can be amplified in vitro given that can undergo to more cell divisions than MSC from adult tissues before reaching senescence. Optimal storage of the cryopreserved umbilical cord tissues or MSC is required for autologous use. In general, easiness of collection and processing make adipose tissue the best source of material suitable for clinical studies aiming at the promotion of tissue healing in the respiratory tract. Collectively, the data acquired so far generally confirm the safety and suggest the occasional clinical efficacy of the delivery of adipose tissue-derived material for the treatment of respiratory-digestive tract fistulas [89]. Nonetheless, there is a strong need to optimize and standardize the protocols to process adipose tissue in order to improve the reproducibility of the procedure. Moreover, the follow-up conditions must be clearly defined to better evaluate the beneficial effects of the treatment.

**Table 3:** Clinical Trials involving tissue- and cell-based therapy approaches to promote fistula and tissue regeneration in the oropharynx

Condition	Intervention	ClinicalTrials.gov Identifier^**1**^
Tracheoesophageal fistula, bronchoesophageal fistula, tracheal fistula	Adipose-derived stromal vascular fraction for aero-digestive fistulae	NCT03792360
Bronchial fistula	Human amniotic epithelial cells for treatment of bronchial fistula	NCT02959333
Bronchopleural fistula	Umbilical cord mesenchymal stem cells for treatment of bronchopleural fistula	NCT02961725
Enterocutaneous fistula	Stromal vascular fraction for treatment of enterocutaneous fistula	NCT01584713
Dysphonia	Innovative treatment for scarred vocal cords by local injection of autologous stromal vascular fraction	NCT02622464
Vocal cord paralysis, unilateral	Injection laryngoplasty using autologous fat enriched with adipose-derived regenerative stem cells	NCT02904824
Hoarseness, dysphonia, aphonia, vocal fold; scar	A study of local administration of autologous bone marrow mesenchymal stromal cells in dysphonic patients with vocal fold scarring	NCT04290182
Vocal fold; scar	Pilot study of bone marrow stem cell treatment of patients with vocal fold scarring	NCT01981330

^1^Source: https://clinicaltrials.gov/, accessed July 2020

**Fig. 5:**
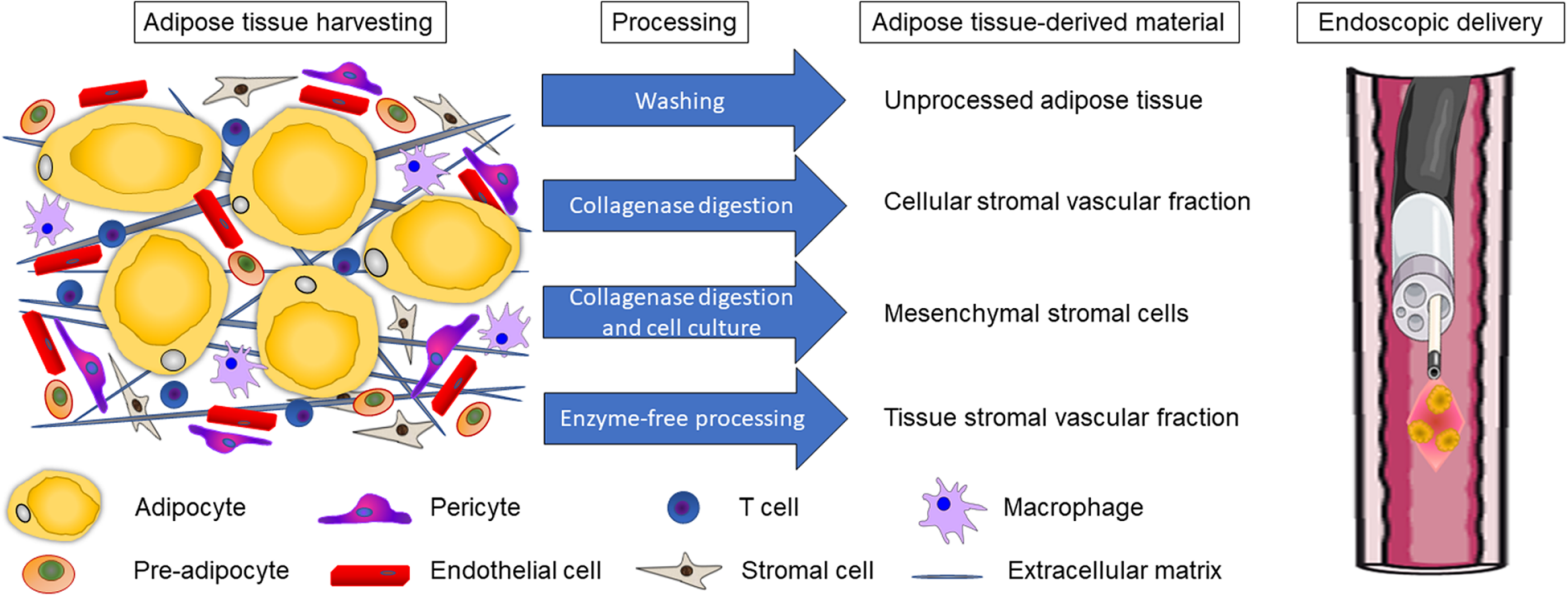
Schematic representation (not in scale) of the therapeutic intervention procedure proposed for the management of respiratory tract fistulas by endoscopic delivery of autologous adipose tissue-derived material. Some templates to create this figure are used/adapted from Servier Medical Art (https://smart.servier.com/), available under a Creative Commons Attribution 3.0 Unported License

The majority of the collected clinical information is based upon studies exploiting administration of unprocessed autologous adipose tissue collected by liposuction (Fig. 5; Tables 1 and 2) [90]. Adipose tissue is mainly composed of adipocytes, which constitute more than 90% of its volume; additional components of the stromal vascular fraction (SVF) include mesenchymal/stromal cells (MSC), preadipocytes, fibroblasts, endothelial cells, vascular smooth muscle cells, resident monocytes/macrophages, and lymphocytes. MSC have a perivascular origin; accordingly, MSC content is higher in vascularized hypodermic adipose tissue [86]. In consideration of the presence within the adipose tissue of cells able to differentiate and to promote tissue regeneration acting in a paracrine fashion [22, 91], fat has been recently reconsidered not only as a simple physical filler for cosmetic surgery procedures, but also as a source of “medicinally signaling cells” [18]. Therefore, as an alternative to or in conjunction with lipotransfer, transplantation of adipose tissue-derived cells has been clinically evaluated for the treatment of a variety of regenerative purposes [25]. In particular, in 2003, Garcia-Olmo firstly reported the effective treatment of rectovaginal fistula in Crohn’s disease by administration of autologous adipose tissue-derived MSC [92]. Subsequently, additional phase I to III clinical trials, collectively enrolling more than 300 Crohn’s patients, have been performed indicating that cell transplantation is safe and effective [26, 27, 29, 93]. Isolation of MSC cells mainly relies on collagenase digestion of the fat collected by lipoaspiration [14]; from the regulatory point of view enzyme-based protocols cannot be considered “minimal manipulation” and therefore the manufacturing procedures are subjected to the regulation applied for the Advanced Therapies Medicinal Products (ATMPs) [94]. Moreover, amplification in culture of MSC requires GMP conditions, is time-consuming, and is associated with high cost and regulatory burden. Therefore, alternative, enzyme-free strategies to obtain a ready-to-use adipose tissue-derived material have been developed [95] and administration of mechanically isolated adipose tissue SVF has been performed for the treatment of different pathologies [96]. In particular, administration of homogenized adipose tissue has been recently proved effective in the treatment of perianal fistulas in patients with Crohn’s disease [97–99], providing a suitable alternative to MSC administration [100].

We believe that the strategy of micro-fragmented adipose tissue transplantation, considered for the promotion of healing of anal fistulas, may be effectively applied also for the management of fistulas affecting the upper esophageal tract (Fig. 5). Administration of micro-fragmented adipose tissue-derived stromal fraction tissue (tSVF) has numerous advantages compared to lipotransfer or administration of MSC: tSVF can be obtained from lipoaspirate by non-enzymatic methods with minimal manipulation; the procedure is rapid and cost-effective and can be performed intra-operatively [95]; in tSVF, the relative number of MSC per tissue volume is higher than in adipose tissue, since adipocytes, red blood cells, oil, and aqueous fractions have been discarded; homogenized tSVF can be precisely administered at the site of tissue damage through a 25-G needle; compared to enzymatically derived SVF, micronized tSVF retains the native extracellular matrix and perivascular structures, reducing induction of anoikis upon transplant [101]; microfat lobules are less prone to oxidative stress compared to unprocessed fat, thus improving graft retention since oxidative damage has a detrimental effect on survival of transplanted cell [102] and adipose tissue [103].

Several preclinical studies assessed the efficacy of MSC therapy for laryngotracheal stenosis [104]. Therapeutic benefit associated to MSC transplant is likely attributable to the secretion of soluble factors and to the release of extracellular vesicles (EVs) [22]. Indeed, administration of conditioned medium collected from MSC cell culture may be instrumental in stimulating resident bronchioalveolar stem cells, supporting tissue regeneration in the respiratory system [105]. Moreover, administration of extracellular vesicles produced by adipose tissue MSC mixed in a thermoresponsive gel has been shown to promote esophageal fistula healing in a porcine model [106]. Accordingly, the delivery of MSC secretome has been proposed as a therapeutic strategy for lung injury and acute and chronic diseases [107, 108]. Notably, micro-fragmented fat has improved paracrine anti-inflammatory, anti-fibrotic, and pro-angiogenic proprieties instrumental for supporting tissue regeneration compared to cultured MSC [109].

Combination of autologous mesenchymal stromal cells and tissue-engineered scaffolds is an interesting and rapidly evolving approach in the regenerative medicine arena, potentially suitable also to support the healing of large-size fistulas and partial or long-segment defects of the esophagus [110]. The ideal scaffold should be biocompatible and biodegradable, with a degradation rate similar to the tissue regeneration time. Placement of tissue-engineered graft has been mostly described in preclinical models: for instance, the use of suture filament embedded with adipose tissue-derived MSC has been used to promote fistula healing in a rat model [111]; promotion of esophageal anastomotic leakage healing has been achieved in rabbits by administration of fibrin scaffolds including MSC [112]; synthetic polyurethane electro-spun grafts seeded with autologous MSC have been tested for esophageal tissue remodeling in pigs [113]. To circumvent possible biocompatibility problems, esophagus-like scaffold-free structures embedded with MSC suitable for esophageal repair have been generated by 3D bioprinting and transplanted in rats [114]. In addition, accurate 3D-printed patient-personalized stent, based on 3D reconstruction of the fistula image, can be created, as assessed for the treatment of enterocutaneous fistulas [115]. Clinical translation of preclinical research on tissue engineering for airways defects has been so far limited but the rapid pace of the technological developments in tissue engineering and in 3D bioprinting can anticipate future therapeutic opportunities [116].

## Conclusions

Respiratory tract fistulas may develop as complications in various surgical interventions, trauma, and accidental foreign body and caustic ingestion or, rarely, may be congenital. Small size (< 2 mm) fistulas generally heal spontaneously, while large caliber fistulas may be associated with severe, life-threatening complications. We reviewed several case reports suggesting that endoscopic local delivery of adipose tissue/MSC may represent a moderately invasive and a relatively safe treatment option, alternative to aggressive surgery, to promote fistula healing. One possible strategy which may provide a further therapeutic advancement could be represented by the delivery of micronized adipose tissue, which can be obtained with minimal manipulation [95]. However, much work remains to be performed before successfully translation of clinically competitive cell- and tissue-based new therapies for respiratory tract fistula healing. In particular, standardization of the procedures, optimization of clinical trial design, and guidance in follow-up analysis are needed in order to assess the long-term occlusion of the fistulas in the treated patients.

## Data Availability

Not applicable.

## Abbreviations

ASC: Adipose tissue-derived stromal cells
ATMPs: Advanced therapies medicinal products
BPF: Bronchopleural fistula
EV: Extracellular vesicles
GMP: Good Manufacturing Practices
MSC: Mesenchymal stromal cells
OAF: Oroantral fistula
PCF: Pharyngocutaneous fistula
SVF: Stromal vascular fraction
TEF: Tracheoesophageal fistula
TEP: Tracheoesophageal puncture
TMF: Tracheomediastinal fistula
VPI: Velopharyngeal insufficiency
